# Selecting Nanobodies Specific for the Epidermal Growth Factor from a Synthetic Nanobody Library

**DOI:** 10.3390/molecules28104043

**Published:** 2023-05-12

**Authors:** Yunier Serrano-Rivero, Julieta Salazar-Uribe, Marcela Rubio-Carrasquilla, Frank Camacho-Casanova, Oliberto Sánchez-Ramos, Alaín González-Pose, Ernesto Moreno

**Affiliations:** 1 Faculty of Basic Sciences, University of Medellin, Medellin 050026, Colombia; 0905yunierserrano@gmail.com (Y.S.-R.); julieta.salazaru@gmail.com (J.S.-U.); marcelaru@yahoo.com (M.R.-C.); 2 Pharmacology Department, School of Biological Sciences, University of Concepcion, Concepcion 4070386, Chile; fcamacho@udec.cl (F.C.-C.); osanchez@udec.cl (O.S.-R.)

**Keywords:** epidermal growth factor, nanobody, synthetic library, phage display

## Abstract

The epidermal growth factor (EGF) is one of the most critical ligands of the EGF receptor (EGFR), a well-known oncogene frequently overexpressed in cancerous cells and an important therapeutic target in cancer. The EGF is the target of a therapeutic vaccine aimed at inducing an anti-EGF antibody response to sequester this molecule from serum. However, strikingly, very few investigations have focused on EGF immunotargeting. Since the use of nanobodies (Nbs) for EGF neutralization may be an effective therapeutic strategy in several types of cancer, in this study, we decided to generate anti-EGF Nbs from a recently constructed, phage-displaying synthetic nanobody library. To our knowledge, this is the first attempt to obtain anti-EGF Nbs from a synthetic library. By applying a selection strategy that uses four different sequential elution steps along with three rounds of selection, we obtained four different EGF-specific Nb clones, and also tested their binding capabilities as recombinant proteins. The obtained results are very encouraging and demonstrate the feasibility of selecting nanobodies against small antigens, such as the EGF, from synthetic libraries.

## 1. Introduction

The human epidermal growth factor (EGF) is a small protein of 53 amino acids, whose fold is structured by three disulfide bonds. It demonstrates potent biological activity in vitro and in vivo by stimulating cell and organ proliferation [1]. Furthermore, the EGF is one of the most critical ligands of the EGF receptor (EGFR) [2], a well-known oncogene frequently overexpressed in cancerous cells, causing cell-cycle deregulation, exacerbated angiogenesis, apoptosis blockade, and tumoral cell migration [3]. It was found very early that EGF detection in carcinomas is closely related to high levels of malignancy [4]. Indeed, EGF signaling is essential for tumor-cell growth for several types of cancer [5,6], and it is linked to the epithelial–mesenchymal transition, in which epithelial cells are transformed into fibroblast-like phenotypes with high motility and invasive properties, contributing to cancer metastasis [7,8]. Therefore, along with the EGFR, the EGF is also considered a potential target for cancer therapy. 

The prevention of cancer using our own immune system against self-antigens is possible due to the observed natural self-reactivity of immune cells against autologous antigens [9]. In the case of the EGF, a self-antibody response against this molecule would diminish the serum availability of EGF, thus reducing EGFR activation and, in consequence, cancer-cell proliferation. Following this active therapeutic approach, a novel anti-EGF vaccine named CIMAvax-EGF was developed. This vaccine is based on a chemical conjugate of EGF and the protein P64k from Neisseria meningitidis, adjuvanted with Montanide ISA 51 VG [10]. The main function of this immunogen is to break the EGF’s self-tolerance, inducing an anti-EGF antibody response, which dramatically reduces the EGF concentration in serum [11]. More than 10 clinical trials have been completed with the therapeutic cancer vaccine CIMAvax-EGF, including phase II, III, and IV trials, demonstrating safety, long-term immunogenicity and a significant effect on survival, with several patients achieving long-term survival after vaccination [10].

An alternative, passive-therapy-based approach for sequestering EGF would involve different antibody formats, particularly, nanobodies (Nbs), which are single-domain antibody fragments derived from the heavy chain antibodies found in camelids [12]. Nbs have several advantageous properties over classical antibodies: small size, high stability and solubility, and easy tailoring for multiple applications. Furthermore, they can achieve high affinities despite the fact that their monomeric binding region displays only three hypervariable loops. Nbs have become a relevant class of biomolecules with multiple applications, especially as diagnostic tools and promising therapeutic agents in cancer and other diseases [13]. They are obtained mostly from immune libraries, constructed from animal immunization with the target antigen [14]. In recent years, however, synthetic nanobody libraries have gained ground as alternative Nb sources, offering several advantages, such as lower costs and faster results [15]. As these libraries are not generated for a specific antigen, they can be used for the selection of nanobodies against numerous antigens, including those with high toxicity or low immunogenicity [15].

A successful example of the use of nanobodies to sequester a circulating cytokine is ozoralizumab—a trivalent anti-TNF nanobody, which was recently approved in Japan for the treatment of rheumatoid arthritis [16]. Strikingly, despite the proven potential of EGF as a cancer target, there are very few investigations focused on EGF immunotargeting. A recent work by Guardiola et al. reported the generation of anti-EGF Nbs using an immune library from alpaca, and showed their ability to block EGFR phosphorylation and produce antitumor effects in vitro [17,18]. To our knowledge, these were the only anti-EGF nanobodies reported before the present study.

Since the use of Nbs for EGF neutralization may be an effective therapeutic strategy in several types of cancer, in this study, we decided to generate anti-EGF nanobodies from our recently constructed synthetic nanobody library, based on the phage-display platform [19]. After three selection rounds, each of which used four different serial elution methods, we obtained about forty positive recombinant phage clones. Twenty of these clones were sequenced, resulting in four different amino-acid sequences. These three distinct nanobodies were produced in BL21 (DE3) and purified by ion exchange (IEC) and immobilized metal affinity (IMAC) chromatography, and their ability to bind to EGF was demonstrated.

## 2. Results and Discussion

### 2.1. Design and Production of a Recombinant EGF Protein

**Figure 1:**
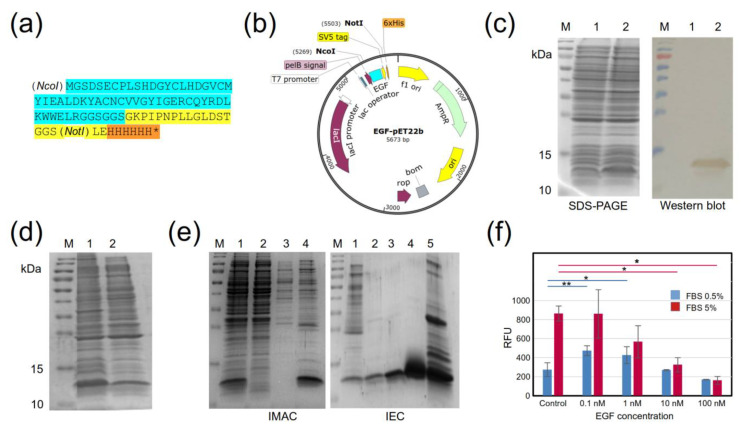
Production of (rEGF) in *E. coli*. (**a**) Amino-acid sequence of rEGF with C-terminal tags: cyan—EGF, yellow—SV5 tag, orange—6xHis tag. (**b**) Scheme of the pET22b-rEGF expression vector. (**c**) SDS-PAGE and Western blot of the production of rEGF in BL21 (DE3). The rEGF was identified using an anti-His tag monoclonal antibody conjugated to HRP. M: molecular-weight marker, Trident Prestained Protein Ladder GTX50875 (GeneTex, USA), 1: untransformed BL21, 2: BL21 transformed with pET22b-rEGF. (**d**) SDS-PAGE of the rEGF-soluble fraction obtained by cell lysis using several freezing/thawing rounds. M: molecular-weight marker, 1: total fraction of BL21 transformed with pET22b-hEGF, 2: soluble fraction of BL21 transformed with pET22b-hEGF. (**e**) SDS-PAGE of the rEGF-purification process. Left panel: first purification stage by IMAC. M: molecular-weight marker, 1: initial sample, 2: unbound proteins, 3: wash (40 mM imidazole), 4: elution (250 mM imidazole). Right panel: second purification stage with IEC. M: molecular-weight marker, 1: initial sample from IMAC elutions, 2, 3, and 4: unbound proteins, 5: elution from the anionic exchanger Bio-Scale Mini Macro-Prep High Q Cartridge (BioRad, Hercules, CA, USA). (**f**) In vitro evaluation of rEGF biological activity in A431 cells. Two FBS concentrations were used: (0.5%—blue bars; and 5%—red bars). Measurements were performed in triplicate. Stars indicate statistically significant differences between experimental conditions (* *p* < 0.05, ** *p* < 0.005).

Firstly, we designed and produced, in-house, a recombinant EGF (rEGF) protein with two tags (SV5 and 6xHis) that confer a more versatile functionality to this molecule (Figure 1a). The rEGF gene was cloned into the bacterial expression vector pET22b (Figure 1b) under the control of strong bacteriophage T7 transcriptional and translational signals, which allow the production of large amounts of recombinant proteins upon induction. The inclusion of the PelB-signal peptide before the rEGF gene allows secretion into the periplasmic compartment, in order to obtain a protein of interest that is soluble after bacterial lysis. The bacterial system (*E. coli* BL21 (DE3)) successfully produced the rEGF, as expected (Figure 1c). After cell lysis, a rEGF fraction remained soluble (Figure 1d), and it was successfully purified by IMAC and IEC (Figure 1e). Although part of the recombinant protein was lost in the different chromatographic steps, we were able to obtain a sufficient amount, with more than 90% purity. The biological activity of the rEGF was assessed in vitro using the human cell line A431, characterized by high EGFR overexpression [20]. The cells were treated with different rEGF concentrations, ranging from 100 pm to 100 nM. Similarly as found in early studies with EGF [21], the lowest applied rEGF concentration (100 pM) stimulated cell growth in cultures with a low concentration (0.5%) of fetal bovine serum (FBS). This effect vanished with higher rEGF concentrations. On the other hand, the expected inhibitory effect of high rEGF concentrations (10 and 100 nM) [21] was markedly observed in A431 cells cultured with 5% FBS (Figure 1f). Having proven the functionality of the in-house-produced rEGF (hereafter called EGF), we proceeded to select EGF binders from our synthetic nanobody library.

### 2.2. Selection of EGF-Binding Phages

As a small protein, EGF presents a relatively small surface area for antibody/nanobody recognition, which presents a challenge in obtaining nanobodies from a synthetic library. We decided to implement a screening procedure based on three selection rounds, each of which was composed of four different types of elution, performed sequentially, using the following: (i) triethylamine (TEA—1), (ii) glycine-HCl (Gly—2), (iii) ultrasound (UltS—3), and (iv) the addition of the TG1 strain to previously washed wells (TG1—4). The aim was to recover binders with different physico-chemical properties that would not be eluted with TEA (a commonly used elution buffer), followed by binders not eluted either with Gly, and so on. After each round, the phages collected from each elution were pulled together for amplification, except for the final round, in which the phages from the different elutions were amplified separately.

The use of successive rounds of selection against the antigen of interest is intended to increase the proportion of antigen-specific phages [14,22]. As in this study, Guardiola et al. performed three selection rounds to obtain EGF-specific phages from an alpaca immune library [17]. In most cases, the phages bound to the target molecule were collected using a single method, which can either be triethylamine (e.g., [17,23,24,25]), glycine [26,27,28], ultrasound (albeit to a lesser extent, such as in [29,30]) and, rarely, infection by the direct addition of cells to the wells [29]. The use of a single elution method may limit phage collection, losing a fraction of phages that remain strongly bound to the immobilized target molecule in the wells. In contrast, in our study, we used four sequential elutions, recovering significant amounts of phages in each of them.

**Figure 2:**
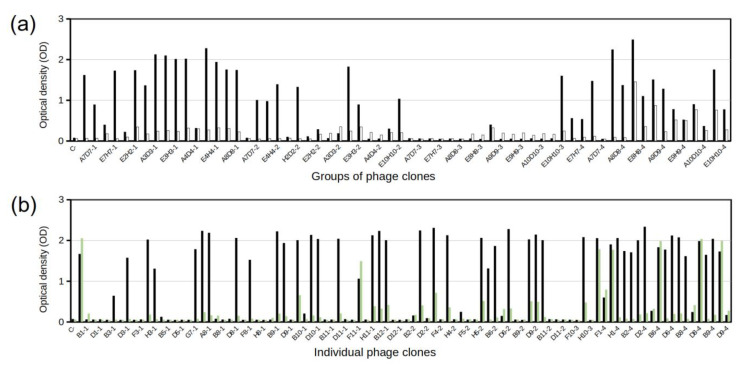
ELISA screening of selected phage clones for binding to EGF. (**a**) Identification of phage groups that bind to EGF (black bars). White bars correspond to background (milk) signals. (**b**) Positive phage groups were individualized and re-screened. Green bars correspond to binding to an unrelated nanobody with the same C-terminal tail as the recombinant EGF. Positivity was defined as OD_450nm_ ≥ 0.8 for EGF binding, and an OD_450nm_ for milk or an unrelated tagged protein three or more times lower than for EGF. The last number in clone names refers to the type of elution: 1—TEA, 2—Gly, 3—UltS—3, 4—TG1.

With the aim of optimizing the screening of clones by ELISA for binding to EGF, we first tested groups of selected clones, each of which consisted of four individual clones (all belonging to the same elution). We evaluated 17, 15, 16, and 16 groups from the TEA, Gly, UltS, and TG1 elutions, respectively. A plate blocked with skimmed milk was included to identify mock-phages. Thus, this assay allowed us to select groups with a high EGF binding signal and low background. Figure 2a shows the ELISA results from this screening. Following the criterion defined in the Methods, a total of 24 positive groups were obtained, which were distributed as follows: TEA-14, Gly-5, UltS-1, and TG1-4. Subsequently, the individual clones from these 24 groups were tested in the same way (Figure 2b), with one important addition. As the recombinant EGF molecule has a C-terminal tail containing SV5 and His tags, we added a third plate coated with a non-related protein with the same C-terminal tail, to discard anti-tag phages. Of the 96 individual clones, 38 met the positivity criteria, and they were distributed by elution, as follows: TEA-16, Gly-1, UltS-1, and TG1-11. A few of these clones, mostly from the TG1 elution, also showed strong anti-tag binding (Figure 2b).

### 2.3. Sequences of Selected Clones

**Figure 3:**
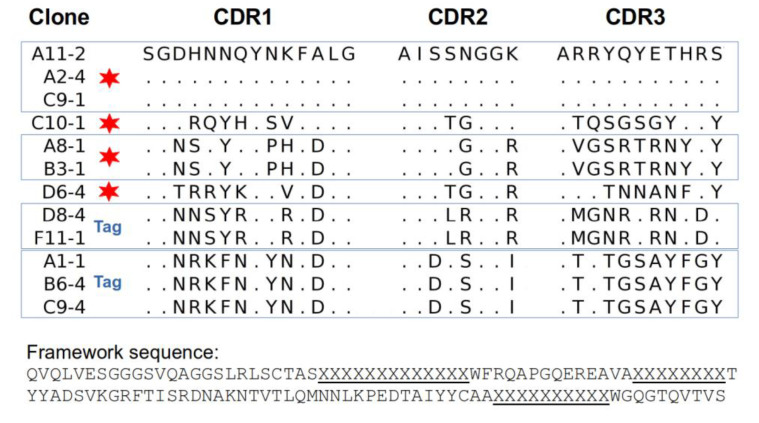
CDR amino-acid sequences of selected clones. Red stars are for EGF binders, while “Tag” in blue is for tag-positive clones. Dots indicate the presence of the same amino acid as in the first sequence in the alignment. The first group of three clones (A11-2, A2-4, and C9-1), from different elutions, are representative of the group of 17 identical clones. The common framework sequence supporting the library is from the camelid nanobody cAbBCII10 [31]). CDRs are underlined, with their amino acids represented with “X”.

Most of the EGF-positive phage clones (21 clones, discarding only a few weaker binders), as well as several of the C-tail tag binders were sent for sequencing. Figure 3 shows the sequences obtained for the CDRs (the framework is common to all the nanobodies derived from the library). The 21 EGF-positive clones corresponded to only four unique Nb sequences, and 17 of the clones were identical. These results are comparable to those obtained by Guardiola et al., who recovered six different anti-EGF clones from an immune library [17].

The use of four different elutions for binder recovery yielded intriguing results. The group of 17 identical clones was composed of phages from three elutions (TEA-8; Gly-2; TG1-7); the identical A8-1/B3-1 pair of clones and the single C10-1 clone were obtained from the TEA elution, whereas the single clone, D6-4, was recovered from the TG1 elution. The mixed origin of the repeated clones obtained after the third selection round might have been associated with the pulling of the four elutions from rounds 1 and 2 for amplification and subsequent inputs for rounds 2 and 3, respectively. On the other hand, the unique D6-4 clone was the result of the sequential application of the four types of elution; otherwise, it would not have been recovered. The five anti-tag phage clones in fact corresponded to two unique Nb sequences. In future screenings, it would be interesting, and possibly more productive, to keep each elution separately through all the selection rounds.

### 2.4. Production of the Four Recombinant Anti-EGF Nanobodies

**Figure 4:**
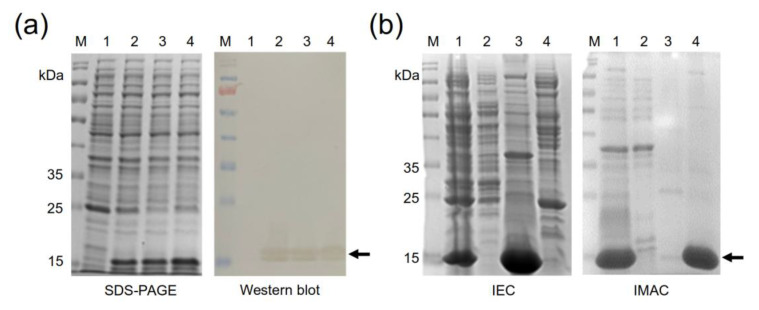
Production and purification of anti-EGF nanobodies. (**a**) SDS-PAGE and Western blot of nanobody production in BL21 (DE3). M: molecular-weight marker, PageRuler Prestained Protein Ladder (Thermo Fisher Scientific, USA), 1: untransformed BL21; BL21 transformed with (2) pET22b-NbA8-1, (3) pET22b-NbA11-2, and (4) pET22b-NbD6-4. The nanobodies were identified using an anti-His tag monoclonal antibody conjugated to HRP. (**b**) SDS-PAGE of the nanobody-purification process. Left panel: first purification stage by IEC. M: molecular-weight marker, 1: initial sample, 2: unbound proteins, 3: elution from the anionic exchanger, Bio-Scale Mini Macro-Prep High Q Cartridge (BioRad, USA), 4: elution from the cationic exchanger CM Sepharose Fast Flow (GE Healthcare, USA). Right panel: second purification stage by IMAC. M: molecular-weight marker, 1: initial sample from IEC, 2: unbound proteins, 3: wash (40 mM imidazole), 4: elution (250 mM Imidazole). Arrow heads indicate the protein bands corresponding to nanobodies A8-1, A11-2, and D6-4.

The four anti-EGF Nbs with different sequences (clones A8-1, A11-2, C10-1, and D6-4) were expressed as recombinant proteins by cloning their genes into pET22b and producing them in *E. coli* BL21 (DE3), which is a commonly used system for nanobody production, with a high level of success [32,33,34]. The recombinant-protein production behaved similarly for the four nanobodies. Upon the induction with IPTG, a majority protein band of approximately 16 kDa was observed in the four induced cultures, while it was absent in the negative control. A Western blot corroborated the observation that all four bands corresponded to recombinant nanobodies (Figure 4a). The design incorporating the signal-peptide PelB allowed the secretion of the produced proteins into the bacterial periplasm, producing a large amount of soluble protein. After production, the four nanobodies were purified by combining two chromatographic methods: ion-exchange chromatography (IEC) followed by immobilized metal-affinity chromatography (IMAC). Usually, a single IMAC purification step is not sufficient to obtain Nbs with a high degree of purity; therefore, at least one additional molecular exclusion or IEC is also used [35], being the combination of IMAC and molecular exclusion chromatography the most commonly used method for Nb purification [25,34,36]. Here, we decided to first carry out an IEC step, which removed more than 50% of the contaminating proteins, followed by IMAC, which yielded recombinant Nbs with about 90% purity (Figure 4b).

### 2.5. Binding Assays for Selected Recombinant Anti-EGF Nanobodies

The recognition of the EGF by the recombinant nanobodies was assessed by indirect ELISA. In a first attempt, we intended to use the SV5 tag in the recombinant EGF molecule for detection, by coating the wells with the recombinant nanobodies, adding the recombinant EGF and then an HRP-conjugated anti-SV5 antibody. This approach, however, yielded very weak signals. We speculate that with such a small molecule as a nanobody, adsorption on a plastic surface would reduce the binding-site availability or affect the conformation of the flexible CDR3 loop.

**Figure 5:**
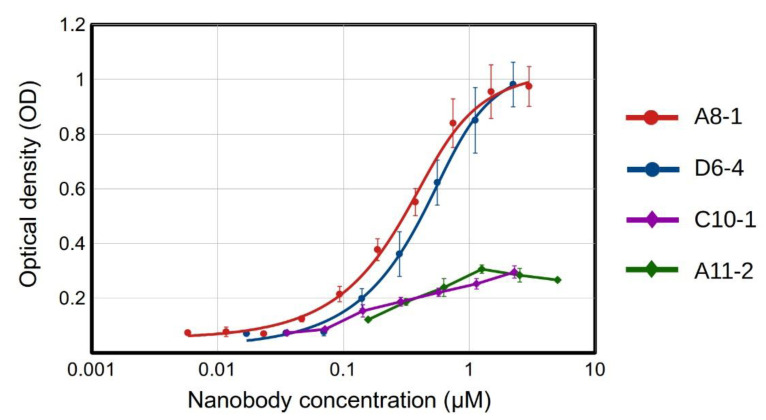
Binding of recombinant nanobodies to EGF by indirect ELISA. Biotinylated nanobodies were detected with streptavidin conjugated to HRP. The red and blue fitting curves were used for KD estimation, for clones A8-1 (KD = (2.4 ± 0.7) × 10^−7^ M) and D6-4 (KD = (3.7 ± 0.8) × 10^−7^ M), respectively. Negative controls (not shown)—BSA coating: OD = 0.11. For all Nbs, we used the maximum tested concentration for the negative controls. Experiments were performed in triplicates.

Since no other tag was available for detection (the His tag is common in both nanobodies and the EGF), we decided to biotinylate the recombinant nanobodies, taking advantage of the three lysine residues found in the framework region. With this method, we successfully detected the expected nanobody–antigen-binding signals, and assessed the dissociation constant (KD) for the two best binders from a titration ELISA [37] (Figure 5). The nanobodies A8-1 and D6-4 showed very similar binding strengths, with KD in the order of 10^−7^ M, while the Nbs A11-2 y C10-1 showed a weaker binding (their KD values could not be assessed).

It is worth noting that while the clone A8-1 has no lysine in its CDRs, D6-4 shows a lysine in CDR1. This lysine could have been affected in the biotinylation procedure, thus abolishing or diminishing the binding capabilities of a fraction of the nanobody molecules. Therefore, the KD for the clone D6-4 might, in fact, be better. The other two clones also displayed lysine residues in their CDRs (two in A11-2, one in CDRs 1 and 2, and one in C10-1 in CDR2), which may have partially affected their binding to EGF. The impairment of the binding capacity of antibodies after biotinylation has been described, and it might be counteracted by establishing an adequate biotin:antibody ratio [38]. The incorporation of biotin into histidine, serine, threonine, and tyrosine residues has also been observed [39]. At least two of these residues are present in the CDRs of the four anti-EGF Nbs, and their possible biotinylation might have exerted a negative effect on the EGF recognition.

Despite these possible drawbacks, the affinities estimated for the clones A8-1 and D6-4 were similar to those measured by Guardiola et al. for five out of their six anti-EGF nanobodies, which showed KD values in the 10^−7^ molar order, while only one clone yielded a KD in the 10^−8^ M order [17]. That is, the anti-EGF nanobodies selected in this work from a synthetic library were comparable in terms of affinity to those obtained from an immunized alpaca. It is worth noting that these KD values are in the same order as the KD of the EGF–EGFR complex, which, in different studies using surface plasmon resonance (SPR) has been found to be within the range of 100–400 nM [40,41,42]. Notably, two of the anti-EGF Nbs obtained by Guardiola et al. were capable of inhibiting the binding of EGF to its receptor and EGFR phosphorylation, with IC50 constants in the micromolar order [17]. They also inhibited cell viability in tumor cells resistant to the EGFR-tyrosine-kinase inhibitor, osimertinib [18].

In this study, we applied a sequential astringent elution method for selecting specific anti-EGF Nb clones from the library, in which the triethylamine solution (suffix 1) was the first elution applied and the addition of TG1 cells (suffix 4) was the last. Therefore, it is reasonable to expect that the nanobodies obtained from the last elution should have a higher binding capacity. Paradoxically, our best binder was obtained from the first elution. This interesting outcome suggests that the nature of the molecular interactions governing nanobody–antigen binding determine the type of elution that detaches the nanobody from the immobilized antigen.

### 2.6. Concluding Remarks

In this study, we obtained four EGF-binding nanobodies from a synthetic library, applying a selection strategy that used four different sequential elution steps along three rounds of selection. To our knowledge, this was the first attempt to obtain anti-EGF nanobodies from a synthetic library. Notably, two of the obtained nanobodies showed KD values similar to those obtained for the Nbs derived from an immune library. It has been shown that it is possible to obtain high-affinity binders from synthetic libraries [15,43]. However, the fact that EGF is a small protein, offering a relatively small surface area for antibody/nanobody recognition, represents a major challenge. Thus, the obtained results are very encouraging. The next step will be to test the neutralizing capabilities of the obtained nanobodies. In future anti-EGF biopannings, as well as in those for other small proteins, we plan to use a different antigen-presentation system, in which the EGF is completely exposed for nanobody recognition, by, for example, using a biotinylated tag for anchoring to a streptavidin base [44]. In this way, we expect to increase the probability of obtaining high-affinity binders from the synthetic library.

## 3. Materials and Methods

### 3.1. Synthetic Library Production

The synthetic nanobody library was designed and constructed by our group as described in [19]. In this study, the library previously cloned into the phagemid pMAC and transformed in the *E. coli* strain SS320 was amplified into the amber suppressor, *E. coli* strain TG1. For this purpose, 300 mL of 2xYT medium containing 100 μg/mL ampicillin was inoculated with the TG1 strain and grown at 37 °C, and at 250 rpm, until an OD_600nm_ of 1.5. The phage library previously obtained in SS320 bacteria was used to transduce TG1 cells at a multiplicity of infection (MOI) of 58 at 37 °C for one hour (30 min static and 30 min at 50 rpm). After spinning at 2000× *g* for 15 min at 4 °C and discarding supernatant, the pellet was resuspended in 300 mL of 2xYT medium with 100 μg/mL ampicillin and incubated at 28 °C, 100 rpm, overnight. Next, 200 mL of the previous culture was added to a final volume of 800 mL of 2xYT medium with 100 μg/mL ampicillin and cultured at 37 °C, 250 rpm until an OD_600nm_ of 2. The culture was transduced with the helper phage M13K07 at a MOI of 25 at 37 °C for one hour (30 min static and 30 min at 50 rpm), and spined at 2000× *g* for 15 min at 4 °C. The pellet was diluted in 500 mL of 2xYT medium containing 100 μg/mL ampicillin, 50 μg/mL kanamycin, and 1 mM isopropyl β-D-1-thiogalactopyranoside (IPTG) for library amplification. After calculating library diversity, recombinant phages were concentrated by precipitation with PEG/NaCl (20% polyethylene glycol 8000 and 2.5 M NaCl) and aliquoted in 10% glycerol.

### 3.2. Production and Purification of a Recombinant EGF Protein

The gene coding the EGF was synthesized and cloned into the pET22b-expression vector (pET22b-EGF) by GenScript (Piscataway, NJ, USA). After transforming chemically competent BL21 (DE3), two inocula of 5 mL, each grown at 37 °C and at 250 rpm, overnight, in 2xYT medium with 100 µg/mL ampicillin were added to two Erlenmeyers of 1 L containing 500 mL each of Saline Minimal Medium M9 (SMM9) (45 mM Na_2_HPO_4_ (Sigma-Aldrich, Burlington, MA, USA), 22 mM KH_2_HPO_4_ (Sigma, Burlington, MA, USA), 19 mM NH_4_Cl (Merck, Rahway, NJ, USA), 8.4 mM NaCl (Sigma-Aldrich, Burlington, MA, USA)), 0.05% yeast extract (Oxoid, Basingstoke, UK), and 100 µg/mL ampicillin. After reaching an OD_600nm_ of 0.5, the gene expression was induced with 25 µM IPTG, and cultures were incubated at 28 °C and at 100 rpm, overnight. Next, cultures were centrifuged at 10,000× *g* for 15 min at 4 °C, and the pellet was resuspended in buffer A (150 mM NaCl, 15 mM Na_2_HPO_4_, pH 7.4) and subjected to five freeze/thaw rounds for cell lysis. Soluble rEGF was obtained in the supernatant after spinning at 10,000× *g* for 15 min at 4 °C. The presence of soluble EGF was verified by sodium dodecyl sulphate–polyacrylamide gel electrophoresis (SDS-PAGE) and Western blot.

For SDS-PAGE, protein samples were diluted in a buffer with beta-mercaptoethanol and run in 15% polyacrylamide and 3% stacking gels. Western blot assay was performed in a 0.2-μm PVDF-transfer membrane (Thermo Fisher Scientific, Waltham, MA, USA) and a semi-dry transfer system Trans-Blot^®^ Turbo™ (Bio-Rad, Hercules, CA, USA) at 0.3 A and 25 V for 30 min. After blocking with 5% skimmed milk in PBS, the membrane was incubated with the HRP anti-6X His tag rabbit polyclonal antibody (ab1187, Abcam, Boston, MA, USA) diluted 1:5000 in the blocking buffer. The reaction was visualized using a DAB substrate kit (Thermo Fisher Scientific, Waltham, MA, USA).

The rEGF was purified in two chromatographic stages. (i) The lysis supernatant containing rEGF was purified by immobilized metal-affinity chromatography (IMAC) by adding 5 mM imidazole to buffer A as equilibrium buffer and the initial sample. After loading the sample, the HisPur Ni-NTA Spin Column (Thermo Fisher Scientific, Waltham, MA, USA) was washed with 25 mM imidazole, and protein was eluted with 250 mM imidazole. Samples, washing, and elution were prepared in buffer A. (ii) For ion-exchange chromatography (IEC), a serial connection of two columns was used. One column featured the weak cation-exchanger CM Sepharose Fast Flow (GE Healthcare, Chicago, IL, USA), and the other was a pre-packed column with the anion exchanger, Bio-Scale Mini Macro-Prep High Q Cartridge (BioRad, Hercules, CA, USA). Both were equilibrated with buffer B (50 mM NaCl, 7 mM Na_2_HPO_4_ (Sigma-Aldrich, Burlington, MA, USA), 83 mM imidazole, pH 8). Samples from IMAC were diluted three times in water and loaded into these columns. After column equilibration with the same buffer B, the anion exchanger was eluted with buffer C (500 mM NaCl, 7 mM Na_2_HPO_4_, pH 5), and the cation exchanger was eluted with the same buffer C at pH 10. The pH of buffers for IEC was carefully adjusted according to the theoretical isoelectric point of rEGF, calculated using Prot-Pi (https://www.protpi.ch/Calculator/ProteinTool (accessed on 10 March 2023)). All fractions were monitored using the purification system, BioLogic LP (BioRad, Hercules, CA, USA). Samples containing the rEGF were diafiltered against PBS (Sigma, USA) in Spin-X^®^ UF concentrators of 5 kDa (Corning, Corning, NY, USA), and analyzed by SDS-PAGE and Western blot, as described above. The rEGF purity was estimated using the analytical software of the iBright 750 Imaging System (Thermo Fisher Scientific, Waltham, MA, USA), and its concentration was determined using the Pierce BCA Protein Assay Kit (Thermo Fisher Scientific, Waltham, MA, USA).

### 3.3. Biological Activity of Recombinant EGF

The EGF activity was determined by an in vitro assay using the A431 cell line. Ninety-six-well plates (2592, Corning, Corning, NY, USA) were used for seeding 2500 cells/well in DMEM plus 0.5% or 5% fetal bovine serum (FBS), which were treated with different EGF concentrations (0.1 nM, 1 nM, 10 nM, and 100 nM) for four days. No EGF was added to negative control. Cell viability was measured with AlamarBlue Cell Viability Reagent (Invitrogen, Waltham, MA, USA) in a Varioskan LUX Multimode Microplate Reader (Thermo Fisher Scientific, Waltham, MA, USA).

### 3.4. Selection of EGF-Binding Phages

Polystyrene high-binding microtiter plates (Costar) were coated with 100 µL of the EGF antigen at 10 µg/mL (eight wells) and incubated overnight at 4 °C. After three washes with phosphate-buffered saline (PBS) plus 0.1% Tween 20 (PBST), wells were blocked with 3% skimmed milk (Sigma-Aldrich, Burlington, MA, USA) in PBS (300 µL/well) for one hour. Wells were washed three times with PBST and incubated at room temperature (RT) for two hours with 100 µL of five-hundred times the library diversity diluted in 0.7% skimmed milk. The PBST was used to perform twenty washes (300 µL/well). Two additional washes were conducted with PBS. Recombinant phage collection was undertaken through sequential steps of four types of elution following neutralization. (i) Triethylamine elution: 0.1 M triethylamine, pH 12.0 (100 µL/well), for 10 min at RT, followed by neutralization with Tris-HCl 1 M pH 7.5 (100 µL/well) for 5 min at RT. (ii) Glycine elution: 0.2 M glycine, pH 2.0 (100 µL/well), for 10 min at RT, followed by neutralization with Tris-HCl 1 M pH 9.1 (100 µL/well) for 5 min at RT. (iii) Ultrasound elution: after adding 100 µL/well of PBS, wells were subjected to ultrasound (40 MHz of potency for 30 min). During the first three elutions, wells were washed twice with PBST and once with PBS (300 µL/well each). (iv) TG1 strain elution: addition of the *E. coli* strain, TG1, in exponential phase of growing (100 µL/well). This elution was performed after the three elutions described above. Simultaneously, the *E. coli* strain, TG1, was transduced with the three elutions and incubated at 37 °C for 30 min. Elutions were seeded in 2xYT plates plus 100 μg/mL ampicillin and incubated at 37 °C, and at 250 rpm, overnight.

### 3.5. Enrichment of Positive Phages

Three biopanning rounds were carried out to enrich the phage pull in EGF binders. For the first round, all colonies from plates obtained from the four elutions were gathered in 15 mL of 2xYT medium with 100 µg/mL ampicillin, and 5 mL was used to inoculate 50 mL of the same medium, which was incubated at 37 °C, and at 250 rpm, overnight. Next, the culture was diluted 10 times (500 mL) and grown until a OD_600nm_ of 2 was reached. Transduction with the helper phage M13KO7 was performed at a multiplicity of infection (MOI) of 20 at 37 °C for one hour (30 min static and 30 min at 50 rpm). Transduced bacteria were spun at 2000× *g* for 15 min and diluted in 500 mL of 2xYT medium with 100 µg/mL ampicillin, 50 µg/mL kanamycin, and 1 mM IPTG for the amplification of recombinant phages at 28 °C, and at 250 rpm, overnight. Amplified phages were concentrated as previously described, for further testing against EGF. The second and third biopanning rounds were conducted in the same way, with a small variation in the final culture volume, from 500 mL to 200 mL.

### 3.6. Indirect ELISA for Detecting Positive Phage Clones

Polystyrene high-binding microtiter plates (Corning, Corning, NY, USA) were coated with 100 µL of EGF at 5 µg/mL and incubated overnight at 4 °C. After washing three times with PBS-0.1% Tween 20 (PBST), wells were blocked with 3% skimmed milk in PBS (300 µL/well) for one hour at RT. Supernatants of individual clones, previously amplified, were added to the plate (200 µL of supernatant) for two hours at RT. The M13KO7 phage was used as negative control. Three washes with PBST were performed, and the anti-M13 antibody (G8P) diluted 1:5000 in 3% skimmed milk was added for one hour at RT, followed by an anti-mouse antibody conjugated with horseradish peroxidase at the same dilution, time, and temperature. Plates were washed three times with PBST, and the reaction was visualized with a TMB kit solution (Thermo Fisher Scientific, Waltham, MA, USA). The reaction was stopped with 2.5 M sulphuric acid, and the absorbance was measured in a microplate reader (ES-20/80, BOECO, Hamburg, Germany) at 450 nm. Plates coated with a recombinant anti-EGFR antibody [45] (carrying the same SV5 and 6xHis tags as the recombinant EGF) at 10 µg/mL and with 3% skimmed milk were used to elude the backgrounds of recombinant phages against the SV5 and histidine tags. Positivity was considered for an OD_450nm_ signal ≥ 0.8 for EGF, and for a signal from milk or the non-related protein that was three or more times lower than for EGF.

### 3.7. Sequencing

The DNA from clones in TG1 were purified using the GenElute Plasmid Miniprep Kit (Sigma-Aldrich, Burlington, MA, USA) and sequenced by Macrogen (Seoul, Korea). Sequences were analyzed using the CLC Genomics Workbench v.21 (QIAGEN Aarhus, Aarhus, Denmark).

### 3.8. Production of Selected Recombinant Anti-EGF Nanobodies

The genes coding the anti-EGF nanobodies were extracted from the pMAC phagemid with the restriction enzymes *NcoI*/*NotI* (New England Biolabs, Ipswich, MA, USA) and cloned in the expression vector pET22b with the same enzymes. Nanobody production was carried out as described above, for EGF. 

### 3.9. Nanobody Purification

The anti-EGF nanobodies were also purified in two chromatographic stages. The first was with IEC, using the same serial connection of two columns described above. Both were equilibrated with half-diluted SMM9 medium. After sample loading in the same equilibrium buffer, each column was eluted separately. as explained previously. Buffer pH was also adjusted based on the theoretical isoelectric point of nanobodies, calculated as described above. The elution from the anion exchanger was diluted three times in water and further purified by IMAC, by adding the same amounts of imidazole as previously described. Fraction monitoring, diafiltering, sample analysis, nanobody purity, and concentration were performed as described above.

### 3.10. Nanobody Biotinylation

Nanobodies were diluted in 25-mM carbonate/bicarbonate buffer at a concentration around 1 mg/mL. Fifty microliters of biotin (H1759, Sigma-Aldrich, Burlington, MA, USA) at 10 mg/mL in DMSO was slowly added to nanobodies. The final molar ratio biotin:nanobody was 40:1. Reaction was stirred for six hours at RT, under protection from light. Free biotin was removed by dialyzing against PBS.

### 3.11. ELISA for Bilotinylated Anti-EGF Nanobodies

Polystyrene high-binding microtiter plates (Corning, Corning, NY, USA) were coated with 100 µL of rEGF at 5 µg/mL and incubated overnight at 4 °C. After washing three times with PBS, 0.1% Tween 20 (PBST), wells were blocked with 3% skimmed milk in PBS (300 µL/well) for 1 h at RT. Biotinylated nanobodies were serially diluted and incubated for one hour at RT. After washing, streptavidin conjugated to HRP (Biotechne R&D Systems, Minneapolis, MN, USA) diluted 1:200 was added for one hour at RT. The reaction was visualized, stopped, and measured, as described above. The KD estimation was carried out by following the method and fitting function described in [37]. A linear regression analysis using this function was performed using the MyCurveFit web server (https://mycurvefit.com/, last accessed on 4 May 2023).

## Data Availability

The data presented in this study are contained in the article tables.

## Sample Availability

Not applicable. The phagemids and molecules used in this work were either purchased or produced in limited amounts to perform the reported experiments only.

## References

[1] Cohen S., Carpenter G. (1975). Human Epidermal Growth Factor: Isolation and Chemical and Biological Properties. Proc. Natl. Acad. Sci. USA.

[2] Singh B., Carpenter G., Coffey R.J. (2016). EGF Receptor Ligands: Recent Advances. F1000Research.

[3] Mitsudomi T. (2014). Molecular Epidemiology of Lung Cancer and Geographic Variations with Special Reference to EGFR Mutations. Transl. Lung Cancer Res..

[4] Tahara E., Sumiyoshi H., Hata J., Yasui W., Taniyama K., Hayashi T., Nagae S., Sakamoto S. (1986). Human Epidermal Growth Factor in Gastric Carcinoma as a Biologic Marker of High Malignancy. Jpn. J. Cancer Res..

[5] Liu T.-C., Jin X., Wang Y., Wang K. (2017). Role of Epidermal Growth Factor Receptor in Lung Cancer and Targeted Therapies. Am. J. Cancer Res..

[6] Cheaito K., Bahmad H.F., Jalloul H., Hadadeh O., Msheik H., El-Hajj A., Mukherji D., Al-Sayegh M., Abou-Kheir W. (2020). Epidermal Growth Factor Is Essential for the Maintenance of Novel Prostate Epithelial Cells Isolated from Patient-Derived Organoids. Front. Cell Dev. Biol..

[7] Kalluri R., Weinberg R.A. (2009). The Basics of Epithelial-Mesenchymal Transition. J. Clin. Investig..

[8] Heerboth S., Housman G., Leary M., Longacre M., Byler S., Lapinska K., Willbanks A., Sarkar S. (2015). EMT and Tumor Metastasis. Clin. Transl. Med..

[9] Gonzalez G., Montero E., Leon K., Cohen I.R., Lage A. (2002). Autoimmunization to Epidermal Growth Factor, a Component of the Immunological Homunculus. Autoimmun. Rev..

[10] Saavedra D., Crombet T. (2017). CIMAvax-EGF: A New Therapeutic Vaccine for Advanced Non-Small Cell Lung Cancer Patients. Front. Immunol..

[11] García B., Neninger E., de la Torre A., Leonard I., Martínez R., Viada C., González G., Mazorra Z., Lage A., Crombet T. (2008). Effective Inhibition of the Epidermal Growth Factor/Epidermal Growth Factor Receptor Binding by Anti–Epidermal Growth Factor Antibodies Is Related to Better Survival in Advanced Non–Small-Cell Lung Cancer Patients Treated with the Epidermal Growth Factor Cancer Vaccine. Clin. Cancer Res..

[12] Muyldermans S. (2013). Nanobodies: Natural Single-Domain Antibodies. Annu. Rev. Biochem..

[13] Morrison C. (2019). Nanobody Approval Gives Domain Antibodies a Boost. Nat. Rev. Drug Discov..

[14] Muyldermans S. (2021). A Guide to: Generation and Design of Nanobodies. FEBS J..

[15] Valdés-Tresanco M.S., Molina-Zapata A., Pose A.G., Moreno E. (2022). Structural Insights into the Design of Synthetic Nanobody Libraries. Molecules.

[16] Keam S.J. (2023). Ozoralizumab: First Approval. Drugs.

[17] Guardiola S., Varese M., Sánchez-Navarro M., Vincke C., Teixidó M., García J., Muyldermans S., Giralt E. (2018). Blocking EGFR Activation with Anti-EGF Nanobodies via Two Distinct Molecular Recognition Mechanisms. Angew. Chem. Int. Ed..

[18] Guardiola S., Sánchez-Navarro M., Rosell R., Giralt E., Codony-Servat J. (2022). Anti-EGF Nanobodies Enhance the Antitumoral Effect of Osimertinib and Overcome Resistance in Non-Small Cell Lung Cancer (NSCLC) Cellular Models. Med. Oncol..

[19] Contreras M.A., Serrano-Rivero Y., González-Pose A., Salazar-Uribe J., Rubio-Carrasquilla M., Soares-Alves M., Parra N.C., Camacho-Casanova F., Sánchez-Ramos O., Moreno E. (2023). Design and Construction of a Synthetic Nanobody Library: Testing Its Potential with a Single Selection Round Strategy. Molecules.

[20] Haigler H., Ash J.F., Singer S.J., Cohen S. (1978). Visualization by Fluorescence of the Binding and Internalization of Epidermal Growth Factor in Human Carcinoma Cells A-431. Proc. Natl. Acad. Sci. USA.

[21] Kawamoto T., Sato J.D., Le A., Polikoff J., Sato G.H., Mendelsohn J. (1983). Growth Stimulation of A431 Cells by Epidermal Growth Factor: Identification of High-Affinity Receptors for Epidermal Growth Factor by an Anti-Receptor Monoclonal Antibody. Proc. Natl. Acad. Sci. USA.

[22] Anand T., Virmani N., Bera B.C., Vaid R.K., Vashisth M., Bardajatya P., Kumar A., Tripathi B.N. (2021). Phage Display Technique as a Tool for Diagnosis and Antibody Selection for Coronaviruses. Curr. Microbiol..

[23] Rojas G., Pupo A., Gómez S., Krengel U., Moreno E. (2013). Engineering the Binding Site of an Antibody against *N*-Glycolyl GM3: From Functional Mapping to Novel Anti-Ganglioside Specificities. ACS Chem. Biol..

[24] Hu Y., Lin J., Wang Y., Wu S., Wu J., Lv H., Ji X., Muyldermans S., Zhang Y., Wang S. (2022). Identification of Serum Ferritin-Specific Nanobodies and Development towards a Diagnostic Immunoassay. Biomolecules.

[25] Hu Y., Zhang C., Lin J., Wang Y., Wu S., Sun Y., Zhang B., Lv H., Ji X., Lu Y. (2023). Selection of Specific Nanobodies against Peanut Allergen through Unbiased Immunization Strategy and the Developed Immuno-Assay. Food Sci. Hum. Wellness.

[26] Kaczmarek J.Z., Skottrup P.D. (2015). Selection and Characterization of Camelid Nanobodies towards Urokinase-Type Plasminogen Activator. Mol. Immunol..

[27] Lakzaei M., Rasaee M.J., Fazaeli A.A., Aminian M. (2019). A Comparison of Three Strategies for Biopanning of Phage-scFv Library against Diphtheria Toxin. J. Cell Physiol..

[28] Kulpakko J., Juusti V., Rannikko A., Hänninen P.E. (2022). Detecting Disease Associated Biomarkers by Luminescence Modulating Phages. Sci. Rep..

[29] Lunder M., Bratkovič T., Urleb U., Kreft S., Štrukelj B. (2008). Ultrasound in Phage Display: A New Approach to Nonspecific Elution. Biotechniques.

[30] Donatan S., Yazici H., Bermek H., Sarikaya M., Tamerler C., Urgen M. (2009). Physical Elution in Phage Display Selection of Inorganic-Binding Peptides. Mater. Sci. Eng. C.

[31] Conrath K.E., Lauwereys M., Galleni M., Matagne A., Frère J.-M., Kinne J., Wyns L., Muyldermans S. (2001). β-Lactamase Inhibitors Derived from Single-Domain Antibody Fragments Elicited in the *Camelidae*. Antimicrob. Agents Chemother..

[32] de Marco A. (2020). Recombinant Expression of Nanobodies and Nanobody-Derived Immunoreagents. Protein Expr. Purif..

[33] Amcheslavsky A., Wallace A.L., Ejemel M., Li Q., McMahon C.T., Stoppato M., Giuntini S., Schiller Z.A., Pondish J.R., Toomey J.R. (2021). Anti-CfaE Nanobodies Provide Broad Cross-Protection against Major Pathogenic Enterotoxigenic Escherichia Coli Strains, with Implications for Vaccine Design. Sci. Rep..

[34] Nagy-Fazekas D., Stráner P., Ecsédi P., Taricska N., Borbély A., Nyitray L., Perczel A. (2023). A Novel Fusion Protein System for the Production of Nanobodies and the SARS-CoV-2 Spike RBD in a Bacterial System. Bioengineering.

[35] Salema V., Fernández L.Á. (2013). High Yield Purification of Nanobodies from the Periplasm of *E. coli* as Fusions with the Maltose Binding Protein. Protein Expr. Purif..

[36] Kariuki C.K., Magez S. (2021). Improving the Yield of Recalcitrant Nanobodies® by Simple Modifications to the Standard Protocol. Protein Expr. Purif..

[37] Eble J.A. (2018). Titration ELISA as a Method to Determine the Dissociation Constant of Receptor Ligand Interaction. J. Vis. Exp..

[38] Cohen L., Walt D.R. (2018). Evaluation of Antibody Biotinylation Approaches for Enhanced Sensitivity of Single Molecule Array (Simoa) Immunoassays. Bioconjug Chem..

[39] Haque M., Forte N., Baker J.R. (2021). Site-Selective Lysine Conjugation Methods and Applications towards Antibody–Drug Conjugates. Chem. Commun..

[40] Wade J.D., Domagala T., Rothacker J., Catimel B., Nice E. (2001). Use of Thiazolidine-Mediated Ligation for Site Specific Biotinylation of Mouse EGF for Biosensor Immobilisation. Lett. Pept. Sci..

[41] Ferguson K.M., Berger M.B., Mendrola J.M., Cho H.-S., Leahy D.J., Lemmon M.A. (2003). EGF Activates Its Receptor by Removing Interactions That Autoinhibit Ectodomain Dimerization. Mol. Cell.

[42] Kuo W.-T., Lin W.-C., Chang K.-C., Huang J.-Y., Yen K.-C., Young I.-C., Sun Y.-J., Lin F.-H. (2015). Quantitative Analysis of Ligand-EGFR Interactions: A Platform for Screening Targeting Molecules. PLoS ONE.

[43] Moutel S., Bery N., Bernard V., Keller L., Lemesre E., De Marco A., Ligat L., Rain J.C., Favre G., Olichon A. (2016). NaLi-H1: A Universal Synthetic Library of Humanized Nanobodies Providing Highly Functional Antibodies and Intrabodies. eLife.

[44] Predonzani A., Arnoldi F., López-Requena A., Burrone O.R. (2008). In Vivosite-Specific Biotinylation of Proteins within the Secretory Pathway Using a Single Vector System. BMC Biotechnol..

[45] Cruz-Pacheco A.F., Monsalve Y., Serrano-Rivero Y., Salazar-Uribe J., Moreno E., Orozco J. (2023). Engineered Synthetic Nanobody-Based Biosensors for Electrochemical Detection of Epidermal Growth Factor Receptor. Chem. Eng. J..

